# Enhancing Urban Mobility with Self-Tuning Fuzzy Logic Controllers for Power-Assisted Bicycles in Smart Cities

**DOI:** 10.3390/s24051552

**Published:** 2024-02-28

**Authors:** Jin-Shyan Lee, Ze-Hua Chen, Yue Hong

**Affiliations:** 1 Department of Electrical Engineering, National Taipei University of Technology, Taipei 10608, Taiwan; michael09@cht.com.tw; 2 Chunghwa Telecom Company, Ltd., Taipei 10048, Taiwan; 3 Department of Mechatronics and Control Engineering, Shenzhen University, Shenzhen 518061, China

**Keywords:** power-assisted bicycles, fuzzy logic controllers, self-tuning modules, energy consumptions

## Abstract

In smart cities, bicycle-sharing systems have become an essential component of the transportation services available in major urban centers around the globe. Due to environmental sustainability, research on the power-assisted control of electric bikes has attracted much attention. Recently, fuzzy logic controllers (FLCs) have been successfully applied to such systems. However, most existing FLC approaches have a fixed fuzzy rule base and cannot adapt to environmental changes, such as different riders and roads. In this paper, a modified FLC, named self-tuning FLC (STFLC), is proposed for power-assisted bicycles. In addition to a typical FLC, the presented scheme adds a rule-tuning module to dynamically adjust the rule base during fuzzy inference processes. Simulation and experimental results indicate that the presented self-tuning module leads to comfortable and safe riding as compared with other approaches. The technique established in this paper is thought to have the potential for broader application in public bicycle-sharing systems utilized by a diverse range of riders.

## 1. Introduction

The safety and social and economic development of modern cities are highly dependent on transportation systems. Within the urban landscapes of major cities worldwide, bicycle-sharing systems have evolved into a vital element of the transportation options offered by smart cities. Through considering environmental conservation aspects, electric bikes (E-bikes) are convenient and efficient for short-distance transportation [1,2,3]. In general, there are two types of E-bikes, pure electric bicycles and power-assisted ones. In a pure electric bicycle, motor power can be generated by driving a hand throttle even when the rider is not pedaling. On the other hand, power-assisted bicycles are also named pedelecs [2], which means a pedal electric cycle. In order to activate the electric motor and receive assistance, the rider must pedal the bicycle. 

In power-assisted bicycles, the power-assisted controller plays a primary role. Two conventional schemes applied to commercial power-assisted bicycles are the constant-assisted power (CAP) and proportion-assisted power (PAP) controllers [4,5,6]. In the CAP system, the rider is supported by the motor with a predefined constant power, while in the PAP system, the motor generates power in accordance with the pedaling torque from the rider. Despite the simplicity of both designs, neither scheme investigates crucial factors such as user profiles and road conditions. In recent studies, there has been a focus on power-assisted control for E-bikes. Kim et al. [7] discussed the limitations of the commonly used power assistant system (PAS) in commercial E-bikes, highlighting its inefficiency stemming from a lack of consideration for riders’ biomechanics. To address this issue, they proposed a new control algorithm that incorporates parameters derived from analyzing human leg kinematics and muscular dynamics, aiming to improve efficiency and enhance the overall riding experience of E-bikes. Aiming to enhance dynamic response and efficiency by considering various external load conditions, Ho et al. [8] introduced a novel torque measurement and control technique for cycling-assisted E-bikes. By analyzing key E-bike riding parameters, they proposed four adaptive motor torque control methods to optimize performance, concluding that wheel acceleration plays a crucial role in determining the E-bike’s synergetic torque performance. In [9], the introduction of an E-bike app, featuring personalized assist levels and a social component, showcased its potential to effortlessly integrate heightened physical activity into users’ daily routines, supported by a successful four-month case study across three European countries. Additionally, ref. [10] proposed an integrated hardware–software system for E-bikes, adept at accurately calculating battery consumption, considering route details and external conditions, and offering precise information on the remaining range to elevate the overall riding experience. In [11], the authors conducted a thorough review exploring the utilization of E-bikes for recovering and monitoring riders’ physical and physiological information. They underscored the effectiveness of E-bikes in promoting physical activity during commuting while emphasizing the essential need for these bikes to be equipped with efficient artificial intelligence systems for data processing.

On the other hand, fuzzy logic concepts have recently been employed in the design of controllers for power-assisted E-bikes. For example, Hsu et al. [12] proposed an approach based on reinforcement learning to manage assisted power for bicycles, with the aim of ensuring riding comfort and safety. Then, the fuzzy Q-learning-assisted motor control was further developed by integrating the fuzzy logic controller (FLC) with Q-learning to improve the riding experience [13]. Moreover, an FLC was devised, utilizing the bicycle’s velocity and cadence as fuzzy input variables, in order to generate assisted motor power [14]. Since bicycle cycling is mostly affected by wind, an FLC-based and wind-aware speed adaptation policy was proposed in [15] to improve the riding experience. Guarisco et al. [16] proposed an FLC for a dual-wheel drive bicycle under different driving conditions to improve riding performance and user comfort. Hung et al. [17] applied an FLC to bicycle speed control and found that the FLC had better stability than a proportional–integral–derivative (PID) controller.

In order to maintain riding comfort and safety, Lee and Jiang [18] introduced a user-adaptive FLC (UAFLC). This advanced approach, equipped with a user-adaptive mechanism, dynamically adjusts the fuzzy parameters in real time for effective regulation. Furthermore, taking into account road profiles, an FLC was extended by incorporating a slope sensor to provide real-time measurements of road conditions [19]. Furthermore, to provide comfort and safety for bicycles assisted by electrical power, Uyar et al. [20] designed and implemented an enhanced FLC-based strategy considering the interaction between humans and bicycles.

As mentioned above, several FLC-based approaches have been presented for controlling power-assisted bicycles. However, in most of the approaches, the fuzzy rule bases are fixed and not adaptive to environmental changes, such as different riders and roads. In this paper, a modified FLC, named self-tuning FLC (STFLC), is proposed for power-assisted bicycles. In the presented scheme, in addition to a typical FLC, a rule-tuning module is added to dynamically adjust the rule base during fuzzy inference processes.

Our main contribution lies in the introduction of a novel STFLC scheme specifically designed for power-assisted bicycles, which, to the best of our knowledge, has not been explored before in this context. Additionally, we conducted both simulations and experiments to evaluate the impact of our proposed STFLC scheme on both powerful and powerless riders. The simulation and experimental results clearly demonstrate that the STFLC scheme we introduced outperforms existing power-assisted control methods, including conventional FLC and UAFLC [18], in terms of performance.

The rest of this paper is organized as follows. Section 2 briefly illustrates the modelling of bicycle dynamics. Then, Section 3 describes the proposed STFLC. Next, Section 4 displays the simulation and experimental results. Finally, Section 5 gives the conclusion.

## 2. Modelling of Bicycle Dynamics

**Figure 1:**
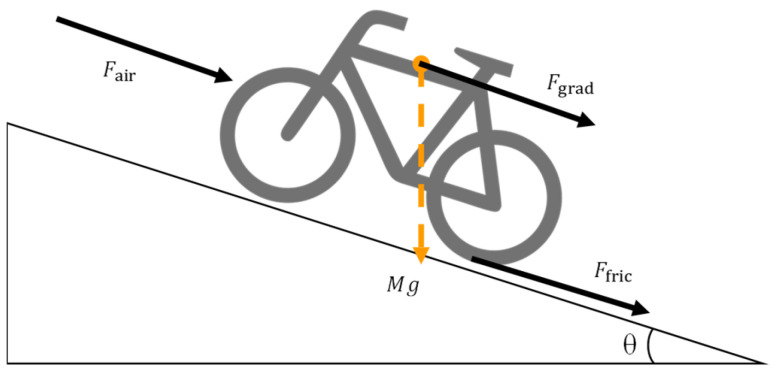
Forces acting on a bicycle during upward motion.

During the riding of power-assisted bicycles, the summation of the pedaling force and power-assisted motor power assisted by the power should be greater than the environmental resistance, so that the bicycle can generate driving force [21]. As riders want to ride their bicycle on uphill slopes, the environmental resistance to be overcome is shown in Figure 1. The environmental resistance includes friction, the slope gradient, and air resistance, as shown in (1), (2), and (3), respectively.

(1) $$F_{\mathrm{fric}}=\mu Mg\cos\theta$$

(2) $$F_{\mathrm{grad}}=Mg\sin\theta$$

(3) $$F_{\mathrm{air}}=1/2\left(C_{d}DA\right)v^{2}$$

The μ is the rolling friction coefficient, M is the summation of rider and bicycle weights (kg), g is the gravitational acceleration ($m/s^{2}$), and θ is the angle between the road surface and the horizontal plane (deg). The $C_{d}$ is the drag coefficient, D is the air density (kg/$m^{3}$), A is the effective area of the wind in front of the rider ($m^{2}$), and v is bicycle velocity (m/s). The above three types of resistance with an expected bicycle velocity $v_{\exp}$ are combined into environmental resistance, as shown in (4).

(4) $$P_{\mathrm{resist}}=\left(F_{\mathrm{fric}}+F_{\mathrm{grad}}+F_{\mathrm{air}}\right)\times v_{\exp}$$

When the rider performs a pedaling action with $P_{\mathrm{pedal}}$, the auxiliary motor immediately provides assisted power as $P_{\mathrm{motor}}$. Then, the total assisted power to be provided to the power-assisted bicycle can be obtained, as shown in (5).

(5) $$P_{\mathrm{total}}=P_{\mathrm{pedal}}+P_{\mathrm{motor}}$$

The bicycle velocity can be mathematically represented as Equation (6).

(6) $$v=P_{\mathrm{total}}/\left(F_{\mathrm{fric}}+F_{\mathrm{grad}}+F_{\mathrm{air}}\right)$$

## 3. Proposed Self-Tuning Fuzzy Logic Controllers

**Figure 2:**
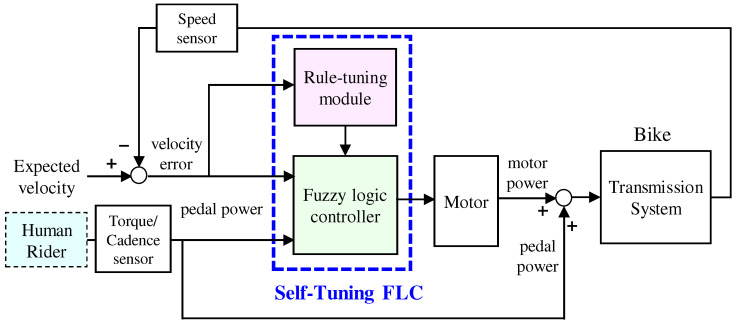
Proposed STFLC scheme for power-assisted bicycles.

A self-tuning FLC, depicted in Figure 2, is presented in this paper. The proposed approach utilizes both pedal power and velocity error as inputs, with assisted motor power as the output. In this scheme, a typical FLC is designed to generate the assisted motor power based on the velocity error and pedal power, considering the desired velocity. The pedal and motor power are then combined and transmitted to the bicycle transmission system. In addition, a rule-tuning module is implemented to dynamically adjust the fuzzy rules, ensuring adequate riding comfort and safety.

### 3.1. Typical Fuzzy Logic Controllers

The inputs of the FLC system are the velocity error and pedal power. After fuzzification, the input value is converted to the membership degree. The rule base completes the fuzzy inference, and then, the degree of membership of the output is converted into the magnitude of the motor power via defuzzification. The velocity error, $\;v_{\mathrm{err}}$, is shown in (7).

(7) $$v_{\mathrm{err}}=v_{\exp}-v$$

**Figure 3:**
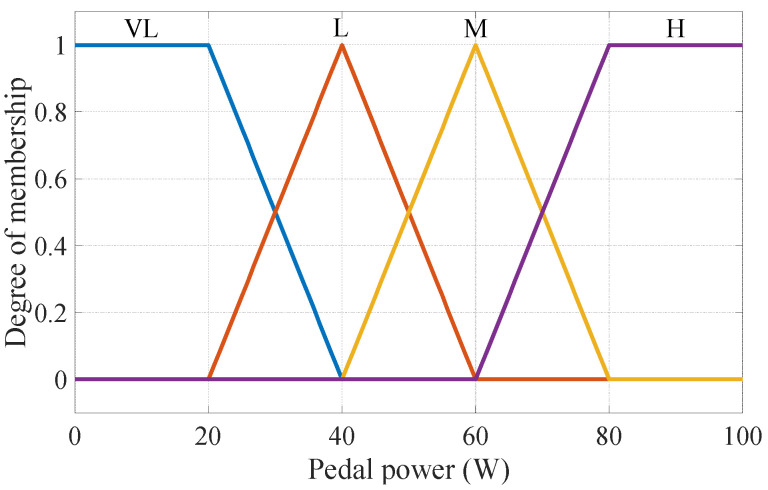
Membership functions of pedal power.

In the fuzzification stage, the inputs are formulated as membership functions. Since the pedal power of the general public is between 0 and 100 W [18], this study takes 0 to 100 W as the range of the membership function. The linguistic values are “Very Low, Low, Medium, High”, and the abbreviations are expressed, respectively, as “VL, L, M, H”; the pedal power membership function is shown in Figure 3.

**Figure 4:**
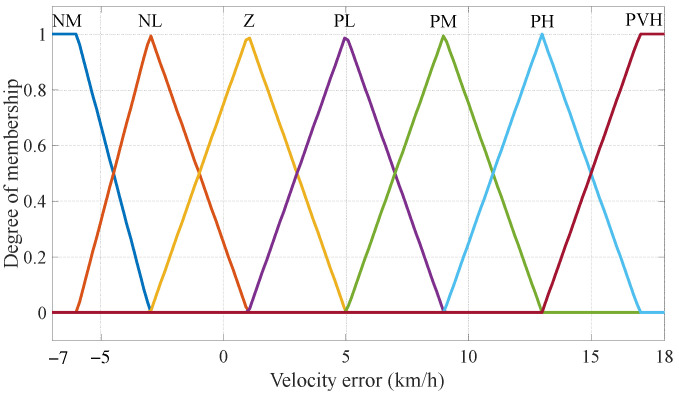
Membership functions of velocity error.

To ensure a comfortable riding experience, it is crucial to maintain the bicycle at a predefined velocity. Based on findings from the literature [4], urban riding typically involves an average and comfortable speed of approximately 18 km/h, which serves as the expected velocity in this study. Moreover, in numerous countries, road regulations dictate that the assist power for E-bikes should be reduced if the bicycle velocity surpasses 25 km/h. Hence, our design encompasses a bicycle velocity range from 0 to 25 km/h, resulting in an error range spanning from −7 to 18 km/h. The linguistic values are “Negative Medium, Negative Low, Zero, Positive Low, Positive Medium, Positive High, Positive Very High”, and the abbreviations are, respectively, expressed as “NM, NL, Z, PL, PM, PH, PVH”. The velocity error membership function is shown in Figure 4.

The rule base consists of two input variables, and the two variables are represented by four and seven memberships. Thus, a total of 28 rules correspond to the output membership function, as represented by Table 1. In general, fuzzy rules could be derived from either experimental data or human heuristics. In this paper, we adopt a heuristic rule generation approach based on the fundamental principle that pedal power and velocity error are directly proportional to motor output power. When pedal power is held constant, a higher velocity error implies a slower vehicle velocity, necessitating an increase in motor output. Conversely, when velocity error remains constant, a greater pedal power suggests uphill riding, requiring an increase in motor output to alleviate the load on the rider.

**Figure 5:**
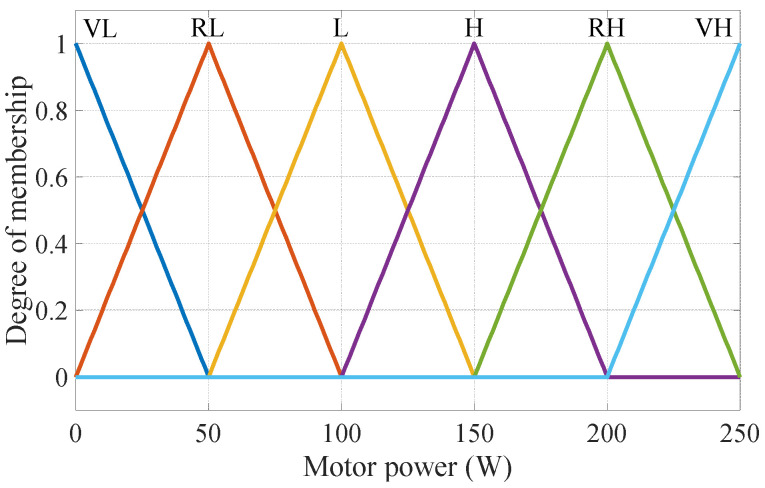
Membership functions of motor power.

This paper uses a 36 V/250 W electric motor. Therefore, the membership function of the motor power output has a minimum value of 0 W and a maximum of 250 W. The linguistic values are “Very Low, Rather Low, Low, High, Rather High, Very High”, and the abbreviations are, respectively, expressed as “VL, RL, L, H, RH, VH”. The motor power membership function is shown in Figure 5.

### 3.2. Rule-Tuning Modules

In general, the system outputs the motor power after inference by the developed FLC via the pedal power and velocity error as inputs in real time. However, different riders have different riding characteristics, and the road conditions in real environments are unpredictable. Therefore, this paper proposes a rule-tuning module, and the fuzzy rules are dynamically adjusted by the data collected during the riding so as to meet different rider characteristics and road conditions.

During riding, the system would record the bicycle velocity, and use the velocity error, that is the $v_{\mathrm{err}}$, as the base to tune the fuzzy rules, as shown in (8). When the velocity error is within a tolerable range, the corresponding rule would remain the same. However, if the error value is outside the range, the rules would be tuned. In our design, the range is set as ±2 km/h. The $∆L_{\mathrm{motor}}^{\left(i\right)}\left(n\right)$ represents the amount of adjustment to the linguistic value of motor power for the corresponding ith rule at the current instant n, and ρ is the learning weight, which is determined from the experiments.

(8) $$∆L_{\mathrm{motor}}^{\left(i\right)}\left(n\right)=\left\{\begin{array}{c} \;0,\;\;\;\;\;\;\;\;\;\;\;\;\;\;\;\mathrm{if}\;|v_{\mathrm{err}}|\leq 2\\ \rho \times v_{\mathrm{err}},\;\;\;\;\;\;\mathrm{if}\;|v_{\mathrm{err}}|>2 \end{array}.\right.$$

Then, the updated linguistic value of the motor power, $L_{\mathrm{motor}}^{\left(i\right)}\left(n+1\right)$, which is used to adjust the corresponding ith rule in the fuzzy rule base, could be obtained from (9).

(9) $$L_{\mathrm{motor}}^{\left(i\right)}\left(n+1\right)=L_{\mathrm{motor}}^{\left(i\right)}\left(n\right)+∆L_{\mathrm{motor}}^{\left(i\right)}\left(n\right)$$

The operation process of the proposed STFLC power-assisted control approach is illustrated in Algorithm 1. Initially, the parameter settings are displayed (lines 1–7), including the rule base *RB*. Once the rider activates the power-assisted mode through an internal command or an external switch, the pedal power and velocity error are continuously monitored. If the rule-tuning module is disabled, the typical FLC is employed with a predefined power range, such as 0–100 W for an average rider (lines 7, 15–16). However, in this particular scenario, the rule-tuning module is automatically enabled (line 5), allowing it to adjust the fuzzy rule base accordingly (lines 8–14).

The proposed rule-tuning module adjusts the linguistic values of the motor power in the employed fuzzy rule. For instance, if the pedal power from the rider is *Low* and the velocity error is *Zero*, the motor power output would be *Low* (see Table 1) as long as the velocity error remains within a tolerable range. However, if the rider encounters unexpected resistance, such as a sudden gust of wind, causing the error to exceed the range, the linguistic value of the motor power may be adjusted to *High* in order to provide the rider with more assisted power.
**Algorithm 1.** Proposed self-tuning fuzzy logic controllers**Input:**           *P*_pedal_: pedal power              *V*_err_: velocity error **Output:**           *P*_motor_: assisted motor power **Function:**          ruleTuningModule(velocity error);                         //return a new rule base;              fuzzyLogicControl(pedal power, velocity error, rule base);                    //return an assisted motor power; **Initialization:** 1.  Initial setting of parameters. 2.  *P*_pedal_ ← 0; 3.  *V*_err_ ← 0; 4.  powerAssistedMode ← False; 5.  ruleTuningMode ← True; 6.  Set the rule base of pedal power and velocity error. 7.  RB← default; //rule base for the ordinary rider (0–100 W) **Main:**
8.  **while** (powerAssistedMode == True) **do** 9.   Read and convert inputs to the pedal power and velocity error. 10.   **if** (ruleTuningMode == True) **then** 11.    Activate the rule tuning module. 12.    Adjust fuzzy rules according to velocity errors. 13.     RB← ruleTuningModule($v_{\mathrm{err}}$); 14.   **end if** 15.   Compute the assisted motor power using the designed FLC. 16.   $P_{\mathrm{moto}r}$ ← fuzzyLogicControl ($P_{\mathrm{pedal}}$, $v_{\mathrm{err}}$, RB); 17.  **end while**

### 3.3. Comparison with Previous Work

In order to maintain riding comfort and safety, Lee and Jiang [18] introduced a user-adaptive FLC (UAFLC) mechanism to dynamically sense pedal power and adjust fuzzy membership parameters with a newly evaluated range of human pedal power. However, their fuzzy rule bases were fixed and not adaptive to environmental changes, such as road conditions. Hence, taking road profiles into account, Dai and Lee [19] added a slope sensor to provide real-time measurements of road conditions and adopted the slope as one of the fuzzy inputs to deduce the proper assisted power. Even though simulation results showed that their approach outperformed the UAFLC in terms of comfortable riding speeds and energy consumption, it required an extra hardware slope sensor and increased the number of fuzzy rules, resulting in a more complicated design.

In this paper, we propose a modified FLC, called the self-tuning FLC (STFLC), specifically designed for power-assisted E-bikes. In addition to a typical FLC, we added a rule-tuning module to dynamically adjust the rule base during fuzzy inference processes. Thus, as compared with the previous UAFLC [18], the rule bases of the STFLC would be adaptive not only to users but also to road conditions. On the other hand, as compared with [19], the proposed STFLC does not require an extra slope sensor to sense road conditions, and the number of fuzzy rules remains the same with the newly added rule-tuning modules. 

We made a significant contribution by introducing a novel STFLC scheme that is specifically designed for power-assisted bicycles. To the best of our knowledge, this scheme has not been explored before in this context. Furthermore, we conducted simulations and experiments to assess the effectiveness of our proposed STFLC scheme on both powerful and powerless riders. The simulation and experimental results show that the STFLC scheme we introduced outperforms existing power-assisted control methods, including conventional FLCs and UAFLCs [18].

## 4. Simulations and Experiments

In the previous work [18], the FLC was compared with the commercial CAP and PAP schemes through a computer simulation. The results indicated that the FLC demonstrated better performance in terms of comfort and safety indices, especially enhancing the CAP system by nearly 40% in comfortability. To evaluate the effectiveness of our proposed approach, we compared the performance of the proposed STFLC with two other power-assisted control approaches, namely the typical FLC and UAFLC [18]. This comparison was carried out for both powerful and powerless riders, with the pedal power ranging from 0 to 140 W and from 0 to 60 W, respectively. The results obtained clearly demonstrate that the proposed STFLC significantly improves the comfort and safety of riding for riders with diverse pedal power levels.

For the simulations, various parameters related to bicycle specifications, environmental conditions, and rider profiles were considered. These parameters are detailed in Table 2.

### 4.1. Performance Indexes

In order to allow the rider to enjoy more comfort when riding a power-assisted bicycle, the rider would like to maintain the desired velocity $v_{\exp}$ during the ride. However, the environment is variable and unpredictable, so a tolerable velocity offset σ was added to define the comfort zone (*CZ*) and *comfortability*, as shown in Equations (10) and (11).

(10) $$\mathrm{CZ}=\{v_{\exp}+\sigma ,\;v_{\exp}-\sigma \}$$

(11) $$Comfortability=\frac{\mathrm{Numbers}\;\mathrm{of}\;\mathrm{velocity}\;\mathrm{within}\;CZ}{\mathrm{Total}\;\mathrm{number}\;\mathrm{of}\;\mathrm{measured}\;\mathrm{velocity}}\times 100\%$$

Comfortability is determined by the ratio of the numbers of velocity falling within the *CZ* to the total measured velocities during the riding distance. According to the literature [4], the expected velocity in urban bicycle riding is typically around 18 km/h, with a velocity offset of 2, implying that the comfort interval ranges from 16 km/h to 20 km/h. For example, 90% comfortability indicates that the bike maintains a velocity within the comfort zone (16–20 km/h) for 90% during the riding distance.

Suppose the rider is riding a power-assisted bicycle. In that case, if the assist force is too large, the acceleration will be too high, causing the rider to reduce the handling of the power-assisted bicycle and increase the chance of a crash. Therefore, according to the literature [12], the safety range (*SZ*) and *safety* are defined by (12) and (13).

(12) $$\mathrm{SZ}=\{-0.4\;m/s^{2},\;0.4\;m/s^{2}\}$$

(13) $$Safety=\frac{\mathrm{Numbers}\;\mathrm{of}\;\mathrm{acceleration}\;\mathrm{within}\;SZ}{\mathrm{Total}\;\mathrm{number}\;\mathrm{of}\;\mathrm{acceleration}}\times 100\%$$

### 4.2. Simulations for Powerful Riders

**Figure 6:**
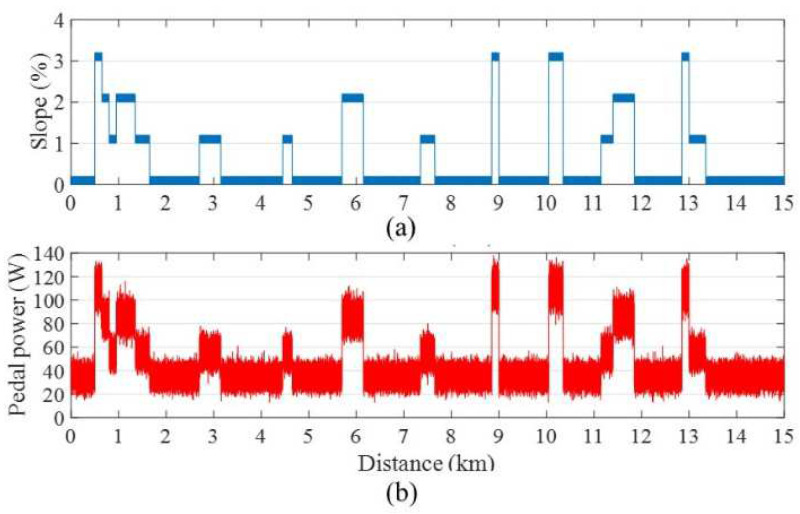
(**a**) Road profile and (**b**) pedal power profile of a powerful rider.

During the experimental phase, the road profile was simulated by incorporating ramps with varying slopes. Specifically, for a 15 km riding distance in an urban area, Figure 6a,b present the planned road profile and pedal power profile of a powerful rider (0–140 W), respectively. The road slope ranges from 0% to 3%, with an additional white Gaussian noise of 0.2% standard deviation. The pedal powers corresponding to different slopes were modeled as a Gaussian distribution [12].

**Figure 7:**
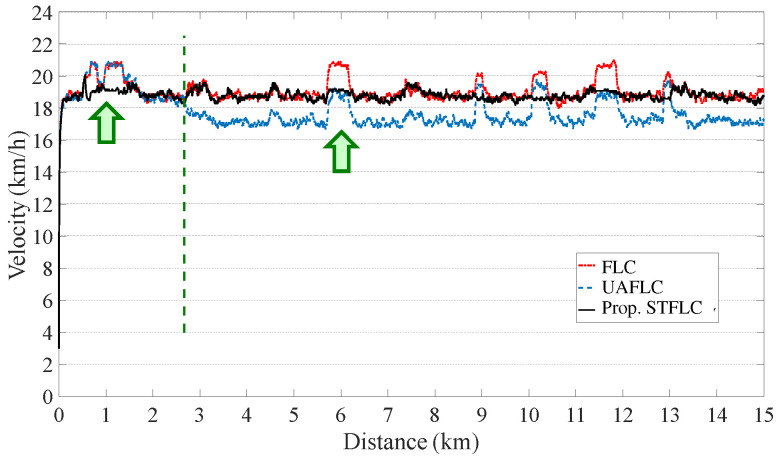
Bicycle velocity using FLC, UAFLC, and proposed STFLC for a powerful rider.

As illustrated in Figure 7, during the initial stage, the velocity curves of the three controllers display similarity as they utilize the typical FLC, which serves as the default option for an ordinary rider. However, after covering a distance of approximately 500 m, the proposed STFLC adjusts its rules based on velocity errors using fuzzy logic for computing the assisted motor power. On the contrary, the UAFLC requires a 2 km adaptation period to determine the adaptive pedal power range for a powerful rider. 

Evidently, both the original FLC and the UAFLC exhibit larger variations in velocity as the slope changes, leading to uncomfortable and unsafe riding experiences. In contrast, the bicycle velocity achieved by the proposed STFLC remains consistently stable, particularly during slope changes (such as at the 6 km mark) and closely aligns with the expected velocity of 18 km/h for urban riding.

In the urban region, Table 3 provides a comparison of the controllers with respect to comfort, safety, and energy consumption for riders with higher power capabilities. As compared with the FLC, the comfortability of the proposed STFLC is greatly improved by 11.24%. Also, as compared with the UAFLC, the comfortability using the proposed STFLC is improved by around 4%. This can be attributed to the rule-tuning module within the STFLC, which dynamically adjusts the rule base to generate assist power that meets the comfort requirement. However, it is worth noting that the STFLC consumes 7% more energy compared to the UAFLC in order to support the rider. Despite this difference in energy consumption, the STFLC significantly improves comfort, as mentioned above. In addition, all three controllers generate excellent safety performance. 

### 4.3. Simulations for Powerless Riders

**Figure 8:**
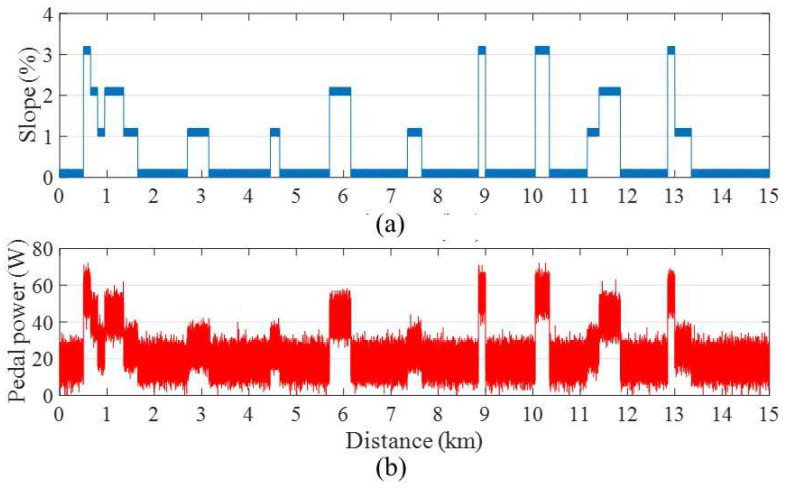
(**a**) Road profile and (**b**) pedal power profile of a powerless rider.

**Figure 9:**
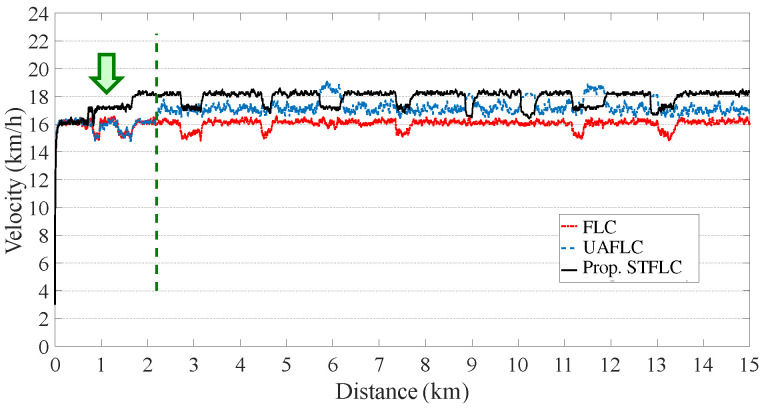
Bicycle velocity using FLC, UAFLC, and proposed STFLC for a powerless rider.

In the case of a powerless rider (0–60 W), Figure 8a,b illustrate the designed road profile and pedal power profile, respectively. Figure 9 demonstrates that when using the typical FLC, the velocity experiences an abrupt drop as the slope increases, particularly around the 3 km mark. This behavior occurs because the original FLC is designed for an ordinary rider and cannot provide adequate motor power to support a powerless rider uphill while maintaining the expected velocity. In contrast, both the proposed STFLC and UAFLC exhibit a more stable bicycle velocity within the comfortable range of 16–20 km/h as the slope increases. Notably, the proposed STFLC achieves a velocity that is much closer to the expected value of 18 km/h compared to the UAFLC.

For powerless users riding in an urban region, Table 4 shows that the proposed STFLC approach outperforms the others with respect to comfortability. As compared with the FLC, the comfortability of the proposed STFLC is greatly improved by 21%. In terms of energy consumption for the powerless rider, the proposed STFLC utilizes approximately 3% more energy to support the rider. However, it also delivers a noticeable improvement of around 5% in terms of comfortability when compared to the UAFLC. In addition, all three controllers generate excellent safety performance. 

### 4.4. Experiment Results

**Figure 10:**
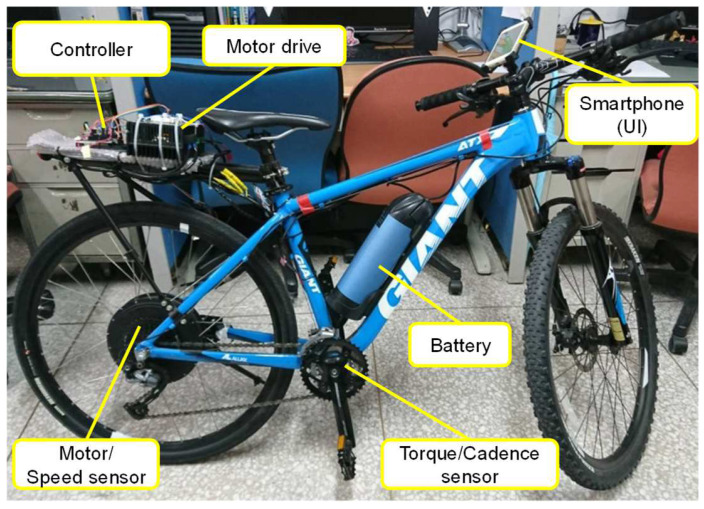
The developed power-assisted E-bike.

The power-assisted E-bike depicted in Figure 10 consists of a controller, a motor drive, a motor with a speed sensor located in the rear wheel hub, a torque and cadence sensor, a battery, and a smartphone serving as a user interface (UI). These components are seamlessly integrated into a conventional bike. When the rider pedals, the controller instructs the motor drive to provide the necessary assisted power every 500 milliseconds, utilizing sensor readings with a sampling time of 60 milliseconds and control algorithms. Additionally, the controller reduces the motor power when the rider stops pedaling or reaches a certain speed, typically 25 km/h. The UI activates the power-assisted system and displays the battery’s state of charge.

Table 5 presents the experimental outcomes for both powerful and powerless riders navigating Taipei City. Evidently, the experimental results exhibit trends parallel to those observed in the simulations. The proposed STFLC surpasses both conventional FLC and UAFLC, demonstrating superior comfort and safety performance. Similarly, regarding energy consumption for powerless riders, the STFLC utilizes approximately 3% more energy to support the rider but achieves a noticeable 4% improvement in comfort compared to the UAFLC.

Despite the experimental results showing lower comfort and safety levels compared to the simulation, the energy consumption is reduced due to the fact that the riding takes place on flat ground. This discrepancy may be also attributed to a realization gap in controller implementation and modeling errors in bike dynamics. Notably, drafting variables, among other parameters like bicycle maintenance, wheel air pressure, and mechanical components (such as the chain), contribute to this variability. Subsequent research endeavors will focus on enhancing the modeling of bike dynamics. Furthermore, while the proposed STFLC method enhances comfort, it concurrently increases energy consumption. Future investigations will include an overhead analysis to explore the potential for reducing energy consumption.

## 5. Conclusions

Ensuring the comfort and safety of riders with varying pedal power across diverse road conditions stands as a primary research focus in the domain of power-assisted E-bikes. This paper introduces a self-tuning fuzzy logic controller (STFLC) specifically tailored for power-assisted E-bikes, with the objective of providing a comfortable and safe riding experience for individuals with diverse capabilities and encountering various road profiles. The simulation and experimental results obtained for both powerful and powerless riders confirm that the proposed STFLC method facilitates a comfortable and safe riding journey while maintaining acceptable energy consumption levels. The approach outlined in this paper is deemed suitable for broader implementation in public bicycle-sharing systems catering to diverse riders. Future directions for research include refining bike dynamics to narrow the gap between simulation and real experiments, as well as conducting an overhead analysis of the proposed approach to minimize energy consumption.

## Figures and Tables

**Table 1:** Fuzzy rules.

	*P*_pedal_	VL	L	M	H
*v*_err_					
NM		VL	VL	RL	L
NL		VL	RL	L	H
Z		RL	L	H	RH
PL		L	H	RH	VH
PM		H	RH	VH	VH
PH		RH	VH	VH	VH
PVH		VH	VH	VH	VH

**Table 2:** Simulation parameters.

Type	Parameter	Value
Bike specifications	Driving type	Real wheel
	Motor type	Brushless rear-hub DC motor
	Motor power	36 V/250 W
	Battery unit	Li-Ion 40 V/11 Ah
	Wheel type	27-inch wheels
	Bike weight	20 kg
Environmental conditions	μ	0.014
	$C_{d}$	0.5
	D	1.184 kg/m^3^
	A	1 m^2^
	μ	0.014
Rider profiles	Rider weight	70 kg
	Pedal power	Ordinary: 0–100 W
		Powerful: 0–140 W
		Powerless: 0–60 W

**Table 3:** Simulations for powerful riders.

	Index	Comfortability (%)	Safety (%)	Energy Consumption (mAh)
Method				
FLC		88.52	99.93	1990.0
UAFLC [18]		95.84	99.92	1800.7
STFLC		99.76	99.91	1934.1

**Table 4:** Simulations for powerless riders.

	Index	Comfortability (%)	Safety (%)	Energy Consumption (mAh)
Method				
FLC		78.24	99.94	2074.8
UAFLC [18]		94.30	99.94	2221.1
STFLC		99.23	99.93	2289.4

**Table 5:** Experiments for both powerful and powerless riders.

	Index	Comfortability (%)	Safety (%)	Energy Consumption (mAh)
Rider/Method				
Powerful rider	FLC	76.34	67.28	1748.2
	UAFLC [18]	80.98	65.66	1533.5
	STFLC	84.57	65.76	1656.2
Powerless rider	FLC	63.08	64.50	1665.6
	UAFLC [18]	73.99	64.29	1749.6
	STFLC	77.72	65.22	1809.3

## Data Availability

The data presented in this study are available on request from the corresponding authors due to privacy restrictions.
